# Targeting DNA Repair and Chromatin Crosstalk in Cancer Therapy

**DOI:** 10.3390/cancers13030381

**Published:** 2021-01-20

**Authors:** Danielle P. Johnson, Mahesh B. Chandrasekharan, Marie Dutreix, Srividya Bhaskara

**Affiliations:** 1 Huntsman Cancer Institute, University of Utah School of Medicine, Salt Lake City, UT 84112, USA; Danielle.Johnson@hci.utah.edu (D.P.J.); mahesh.chandrasekharan@hci.utah.edu (M.B.C.); 2 Institut Curie, CNRS, INSERM, University Centre, 91405 Orsay, France

**Keywords:** DNA repair, cancer therapy, repair inhibition, histone deacetylases

## Abstract

**Simple Summary:**

Targeting aberrant DNA repair in cancers in addition to transcription and replication is an area of interest for cancer researchers. Inhibition of DNA repair selectively in cancer cells leads to cytotoxic or cytostatic effects and overcomes survival advantages imparted by chromosomal translocations or mutations. In this review, we highlight the relevance of DNA repair-linked events in developmental diseases and cancers and also discuss mechanisms to overcome these events that participate in different cellular processes.

**Abstract:**

Aberrant DNA repair pathways that underlie developmental diseases and cancers are potential targets for therapeutic intervention. Targeting DNA repair signal effectors, modulators and checkpoint proteins, and utilizing the synthetic lethality phenomena has led to seminal discoveries. Efforts to efficiently translate the basic findings to the clinic are currently underway. Chromatin modulation is an integral part of DNA repair cascades and an emerging field of investigation. Here, we discuss some of the key advancements made in DNA repair-based therapeutics and what is known regarding crosstalk between chromatin and repair pathways during various cellular processes, with an emphasis on cancer.

## 1. Introduction

Therapeutic targeting strategies for developmental diseases and cancers are being designed independent and dependent of chromatin mechanisms. Aberrant levels of DNA repair and histone modifications result in an imbalance in the genome and epigenetic equilibrium. It is critical to assess the advantages and drawbacks of targeting a single pathway or multiple signaling mechanisms that control genome stability in order to achieve maximum long-term potency with minimal side-effects.

## 2. DNA Repair Is Dysfunctional in Certain Developmental Diseases and Cancers

The mammalian genome is constantly challenged by both endogenous and exogenous agents that cause DNA lesions capable of inhibiting replication or transcription, which can lead to developmental defects or cancers. Congenital defects within the DNA repair networks themselves can additionally lead to cancer. Defects such as these have provided insights into how the DNA repair and damage response pathways work, especially when novel components are identified. Genomic instability is an enabling hallmark of cancer, and defects in DNA repair facilitate the acquisition of genetic events that ultimately promote oncogenic transformation [1]. This knowledge has led to the investigation and use of targeted inhibitors of various repair pathways to sensitize cancer cells to DNA-damaging agents, including conventional chemotherapies and radiation treatments. Disruption of DNA repair mechanisms by targeting proteins that are mutated in developmental diseases may also block cancer development. For instance, Werner syndrome, which is an autosomal recessive progeroid syndrome that is caused by a mutation in the gene encoding repair factor Werner RecQ like helicase (WRN), is associated with defects in DNA repair and telomere maintenance [2,3]. Bloom syndrome is another classical example of a developmental disease that is caused by a mutation in *BLM*, which encodes a DNA repair-linked helicase gene that predisposes patients to develop leukemia and lymphoma [4,5]. Whereas defects in proteins such as these can lead to malignancies, they are also potential druggable targets for other cancers, particularly cancers with heightened DNA repair. Thus, small-molecule inhibition of DNA repair linked to WRN and BLM helicases are currently being tested in the clinic [6,7].

## 3. MRN and ATM/ATR Repair Signal Recognition Factors as Cancer Treatment Targets

The MRE11-RAD50-NBS1 complex (MRN) is one of the first complexes to arrive at sites of double-stranded breaks (DSBs) in genomic DNA [8,9,10,11]. The MRN complex plays a vital role in the downstream signaling involved in the DNA damage response (DDR), including DNA repair, cell-cycle check points, and DNA replication [8,9,10,11]. Given its importance in the DDR, it is not surprising that pathogenic mutations in genes that encode members of the MRN complex can lead to diseases that exhibit DDR impairment and radio sensitivity [12]. For example, *Mre11a* mutations result in a mild form of ataxia-telangiectasia (A-T) that clinically manifests with cerebellar ataxia and oculocutaneous telangiectasia but not cancer development [10]. Nijmegen breakage syndrome is the direct result of mutations in *NBS1*. Patients have microcephaly, combined immunodeficiency, growth retardation, and a predisposition to lymphoma [10,12]. In 2009, a disorder in which compound heterozygous *RAD50* mutations occurred was described for the first time. This patient exhibited microcephaly and growth retardation consistent with Nijmegen breakage syndrome, but no immunodeficiency or lymphoma [13]. This novel prognosis was termed Nijmegen breakage syndrome-like disorder. Cells taken from patients with any of these three syndromes are sensitive to ionizing radiation and exhibit reduced activity of downstream repair factors such as ATM, even though not all of these disorders necessarily predispose patients to malignancies.

A-T is one of the most well-known examples of a congenital defect of the DNA repair pathway. A-T was first described nearly 100 years ago, but the defective gene, *ATM*, was not identified until nearly 70 years later [14]. A-T is characterized by cerebellar ataxia, oculocutaneous telangiectasia, increased incidence of lymphoid tumors, and radio-sensitivity, among other abnormalities [14]. The *ATM* gene codes for a Ser/Thr protein kinase that is involved in the DDR. ATM associates with the MRN complex at sites of double-strand breaks (DSBs) during the early stages of DDR, and the phosphorylation of ATM begins a signaling cascade that activates or recruits further repair proteins and that triggers cell-cycle check point responses [15]. Patients with A-T have an impaired DDR that causes radio-sensitivity and increases cancer risk, specifically for lymphoid cancers [14].

Although mutations within *ATM* can predispose A-T patients to malignancies, the diverse roles of ATM in the DDR makes it a potential druggable target for cancer patients who have radio-resistant tumors [16]. Glioblastoma is the most malignant form of glioma, and it is characterized by a high rate of radio- and chemo-resistance, due to its “addiction” to DNA repair. ATM inhibition should, therefore, be a viable therapeutic strategy for glioblastoma. In fact, there is currently a phase I clinical trial underway with the ATM inhibitor AZD1390 in glioblastoma patients (NCT03423628) [17]. This trial will explore safety and efficacy of ATM inhibition in combination with radiation therapy for the treatment of glioblastoma.

Ataxia-telangiectasia and Rad3-related protein (ATR) is another Ser/Thr protein kinase similar to ATM that is involved in DDR. ATR is activated by a broad range of DNA damage signals [18]. The localization of ATR to sites of damage is dependent on its interaction with ATR interacting protein (ATRIP) and replication protein A (RPA) coated ssDNA [19,20]. In addition, DSB resection mediated by the MRN complex to create ssDNA is also important for ATR activation [21]. ATR is mutated in a number of cancers and because of its key role in replication, the resulting replication stress can drive cancer development as reported before [22,23,24]. ATR works with downstream checkpoint protein kinase 1 (CHK1) to inhibit DNA replication, but recent studies have demonstrated that ATR modulates replication even in the absence of DNA damage [25]. ATR inhibitors could also be beneficial in liquid cancers that rely heavily on ATR functions for DNA repair, such as chronic myelomonocytic leukemia (CMML) or myelodysplastic syndrome (MDS) and in fact, ATR inhibitor AZD6738 is currently under phase I clinical trial for use in these malignancies (NCT03770429) for progressive MDS or CMML.

Mutations in ATR have also been linked to the rare Seckel syndrome type 1 (ATR-Seckel) (MIM #210600). Seckel syndrome is a heterogeneous developmental disorder derived from a number of different mutations in cell cycle regulatory genes, such as ATR, CEP152 and CENPJ [26,27]. Common characteristics of Seckel syndrome include dwarfism, mental retardation, “beak-like” facial features, and intrauterine growth retardation. Similar to other developmental disorders described above, ATR-Seckel cells demonstrate impaired DNA repair but a link to cancer predisposition has not been well established in these patients [28].

Even though both ATM and ATR are involved in the DDR, they have distinct roles when damage occurs. ATM is typically associated with DSBs particularly those induced by irradiation, but ATR can be stimulated in a variety of DNA damage-inducing conditions as well as during replication stress [29,30,31]. ATM is not required for cellular viability, whereas ATR is critical, and loss of ATR function results in embryonic lethality in mouse models [32]. Confounding these separate mechanisms is the evidence of crosstalk between the two proteins. ATM and ATR may directly or indirectly activate one another. For instance, ATR can phosphorylate histone H2AX during replication stress, which can then recruit ATM to stressed replication forks [33], and ATM can enhance DNA end resection, thus promoting the activation of ATR [30,34]. Additionally, there is evidence that ATM and ATR can function redundantly during the DDR when one of them is absent [35,36]. This suggests that in some instances, it may actually be beneficial to target both kinases, and if one kinase is deficient, targeting of the other may be advantageous.

The DNA-dependent protein kinase (DNA-PK) complex is another Ser/Thr protein kinase similar to ATM that is involved in the DDR, it is the case for ATR, DNA-PK has never been associated with a developmental disease in humans. However, numerous clinical studies have reported evidence correlating aberrant DNA-PK status or activity with cancer onset, progression, and responses to therapeutic modalities. Notably, multiple studies have established the roles of DNA-PK outside DDR network, corroborating its functions as a pleiotropic complex involved in transcriptional programs that operate in biologic processes such as the epithelial to mesenchymal transition, hypoxia, metabolism, nuclear receptor signaling, and inflammatory responses [37]. DNA-PK is an obvious therapeutic target in cancer, and data pertaining to various pharmacological approaches have been published, largely in the context of combination with DNA-damaging agents that act by causing DSBs [38]. DNA-PK inhibitors M3814, also known as MSC2490484A (NCT02516813) and CC-122 are currently being evaluated for the treatment of advanced solid tumors, leukemia, or lymphoma either alone or in association with radiotherapy or chemotherapy [39,40,41].

## 4. Targeting Cell-Cycle Checkpoints in Cancer

To avoid permanent damage to DNA and allow time for repair, normal cells integrate the DDR network into cell-cycle control via downstream checkpoint signaling. ATM and ATR are two of the master regulators of the checkpoint pathways. The complex of ATM and the check-point kinase CHK2 responds to DSBs to induce a G1 arrest, whereas ATR-CHK1 triggers S and G2 arrest [42]. Most tumors lack an intact G1 phase checkpoint response and rely on S and G2 checkpoints for repair and survival, which are regulated by CHK1 activity [43]. Moreover, increased checkpoint activities are often seen in human cancer cells that develop resistance to chemotherapy or radiotherapy. Inhibition of CHK1 activity impairs repair and promotes tumor cell death [44]. In spite of that, most checkpoint inhibitors, even when used along with inhibitors of DNA repair, have failed in clinical trials [45]. One reason for this failure is the absence of a thorough understanding of the mechanisms of action and the lack of appropriate biomarkers for assessment of upstream and downstream responses to defective DNA repair components in the context of particular cancers. Targeting checkpoint responses to overcome radio-resistance and improve therapeutic outcomes in brain cancer has been a huge initiative but with only moderate success so far [46,47].

## 5. The Use of Synthetic Lethality in Cancer Therapeutics and Its Limitations

Loss of function of a particular DDR pathway can make cancer cells depend on compensatory pathways, and thus targeting more than one DDR pathway can make cancer cells accumulate synthetic lethality-driven DNA damage. Upstream of ATM is PARP1, which synthesizes poly-ADP ribose (PAR) and transfers these moieties to proteins. PARP1 activity is essential for the recruitment of the MRN complex to DSBs [48]. The PARP inhibitor Olaparib is the first clinically approved DNA repair inhibitor designed to utilize this synthetic lethality property for cancer therapy [49]. The *BRCA1/2* genes are the most commonly mutated genes in hereditary breast and ovarian cancers [50]. *BRCA1/2* are tumor suppressor genes that control homologous recombination, and *BRCA1/2*-mutant cells are sensitive to PARP1 inhibition [51]. PARP inhibitors sensitize cancer cells to both chemo- and radio-therapy and BRCA1/2-deficient cells are 1000 times more sensitive to PARP inhibitors than wild-type cells [51,52,53]. Unfortunately, resistance emerges even with a combination of PARP and ATM/ATR inhibitors. Additionally, there are safety issues associated with long-term treatment with PARP inhibitors. On the brighter side, a third generation PARP inhibitor, rucaparib, is being tested in a randomized phase III clinical trial (NCT01968213) following promising results in earlier stages of clinical development [52,54].

As alluded to above, ATM/ATR inhibition is another viable target for synthetic lethality. While, ATR loss of function is rare in cancers, ATR inhibition may be particularly potent in cancer cells with other, specific mutations, such as in ATM, when compared to normal cells. Clinical trials with the ATR inhibitor AZD6738 intend to do just this. One trial aims to induce synthetic lethality in cells with ATM deficiencies, specifically chronic lymphocytic leukemias (CLL) that are ATM deficient (NCT01955668). This study has been completed but to date there are no published results. Another clinical trial is aimed at targeting ATM deficient advanced lung adenocarcinomas as well as high grade ovarian cancers that harbor BRCA1/2 mutations using a combination of AZD6738 and carboplatin (NCT02264678) [55]. This trial as well as others are additionally examining the effects of AZD6738 alone or in combination with PARP inhibitor to treat a number of solid tumors, including ATM deficient gastric cancer (NCT03682289, NCT03462342) [55]. All of these studies are underway with limited results published at this time.

Synthetic lethality may also be induced by MRE11 or RAD50 inhibition. The viability of this strategy is supported by the findings that subjects with A-T-like disorder and Nijmegen breakage syndrome-like disorder have impaired repair via reduced ATM activity but do not appear to have higher risks of malignancy. Mirin is an MRE11 inhibitor [56] that affects the entire MRN complex and subsequent ATM signaling. MRE11 mediates MYCN protooncogene dependent replication stress [56], and MYCN activation is an oncogenic driver and a marker of poor prognosis. Treatment of *MYCN*-amplified neuroblastoma mouse models with mirin resulted in a significant reduction in tumor growth [56]. Despite promising in vivo studies, mirin and mirin analogs have not proceeded beyond pre-clinical stages of development, and therefore advancement is necessary in this area of clinical investigation.

## 6. Challenges Associated with Targeted Therapies and a Novel Strategy to Achieve Pan DNA Repair Inhibition

The unique requirements of DNA repair pathways have been exploited extensively in the search for treatments for both solid and liquid cancers. It is critical to stratify patients based on the DDR status of the tumor under treatment. DNA lesions must be repaired effectively during all phases of cell cycle and eukaryotic cells have developed complex DDR networks that function differently in various cell-cycle stages. The key to any repair-targeting strategy is that the mechanistic differences between normal and cancer cells must be evaluated to avoid toxic side effects that might arise from inhibiting DNA repair in normal cells (Table 1).

**Figure 1:**
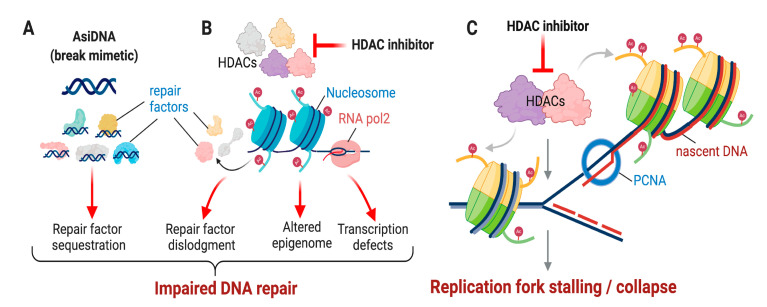
Mechanisms by which AsiDNA treatment and histone deacetylase (HDAC) inhibition alter genome stability during repair and replication in cancer cells. (**A**) AsiDNA acts as a DNA mimetic to sequester DNA repair factors. (**B**) Multiple ways by which HDAC inhibition can impair DNA repair. Stalled transcribing RNA polymerase II (pol2) and resulting RNA-DNA hybrid or R-loop are shown. (**C**) Replication defects caused by HDAC inhibition. Newly replicated or nascent DNA in leading or lagging strand is shown in maroon, and the non-replicated or old DNA is shown in light blue.

A novel strategy is to target the DDR as a whole, rather than inhibiting just one sensor or kinase or even using a cocktail of inhibitors of specific factors. AsiDNA is a first-in-class DDR activator that sequesters repair factors away from break sites to create an artificial damage response signal selectively in cancer cells [67] (Figure 1A). AsiDNA is a double-stranded DNA oligonucleotide that acts as a break mimetic to impair multiple DDR pathways in liquid as well as solid tumor cells [67]. This global inhibition of repair pathways causes a prolonged retention of DNA damage signals in melanoma and glioma cells when used along with radiation [66,68]. A first-in-human phase 1/2a trial with AsiDNA (DNA repair inhibitor and irradiation on melanoma (DRIIM), NCT01469455) in patients with metastatic melanoma demonstrated the safety of local administration of this compound [71]. Additionally, no maximum-tolerated dose was identified, and AsiDNA induced tumor regression that correlated with systemic exposure [71,72]. Induction of DNA damage in surrounding normal tissues is a major issue with radiation. Our group demonstrated that AsiDNA acts as a radiosensitizer in tumors but does not enhance the toxicity of the radiation in surrounding the healthy tissues. This property was demonstrated in pediatric models of brain tumors setting the stage for a clinical trial to treat recurring glioma in children using AsiDNA in association with radiotherapy (NCT03579628) [70]. AsiDNA in combination with carboplatin (causes DNA lesions via formation of adducts) and paclitaxel (stabilizes microtubules to block the cell cycle at the G2/M phase) is well tolerated (NCT03579628) [73,74].

## 7. Chromatin Interactions Associated with Repair, Transcription, and Replication and Implications for Cancer Therapy

Chromatin organization has a profound effect on DNA repair-modulated dynamic changes in histone modifications and vice versa. A number of histone modifications, including histone phosphorylation, ubiquitylation, acetylation, methylation, and sumoylation, that occur in the vicinity of breaks are associated with the DDR and DNA repair [75]. A majority of these modifications are also involved in other cellular processes such as transcription and replication that interconnect to ensure proper cellular function and timely genome maintenance. We will not list all the histone modifications and their functions in DNA repair here. Instead, we will focus on how dysfunctional crosstalk between DNA repair, transcription, and replication poses a threat to genome stability in cancer cells in a chromatin-dependent manner.

Conventionally, analysis of the DDR has focused on the damage itself or response proteins and their modifications rather than the chromatin landscape. It is imperative to understand the dynamics of chromatin, in particular histone modifications, as chromatin organization rapidly changes in the context of DNA damage [76]. One of the first studies to examine chromatin in this context was a massive screen of histone modifications in U2OS and HeLa cells [77]. This screen revealed a global reduction in H3K9ac and H3K56ac when cells were exposed to DNA-damaging agents [77]. These modifications are particularly interesting as they have been observed at the promotors of actively transcribed genes, and sites of transcription are potential sites of DNA damage [78].

DNA–RNA hybrids (R-loops) are formed when the replication machinery collides with the transcriptional machinery [79]. R-loops occur transiently during normal replication but are stable when the DNA and RNA hybrids remain unresolved and paired for up to 2000 base pairs [80]. At R-loops, DSBs, fork collapse, or incomplete replication can result [81]. The structure of the R-loop itself may also make the DNA more prone to breakage due to flaps that are formed on either end of the R-loop [82]. Replication stress and DNA damage caused by R-loops then activate both ATR and ATM signaling pathways [79,80].

The Fanconi anemia (FA) pathway resolves R-loops by stabilizing the replication fork and by activating enzymes such as RNase H that resolve the RNA-DNA hybrids [83,84]. Interestingly, our research has shown that inhibition of class I histone deacetylases (HDACs) can lead to the formation of R-loops. In leukemia patient-derived xenograft mouse models that were treated with HDAC inhibitors, we observed an accumulation of R-loops within the bone marrow [85]. Mechanistically, we showed that inhibition of class I HDACs decreases the chromatin-bound levels of MRN complex components Mre11-RAD50 and NBS1 [85]. Interestingly, the MRN complex also activates the Fanconi anemia pathway to suppress R-loop formation. Therefore, HDAC inhibitors not only increase R-loop formation but also prohibit their repair by altering the Fanconi anemia pathway and other core repair components, such as the MRN complex. Whether alternative mechanisms mediated by HDACs control timely resolution of R-loops in a cell-cycle-dependent manner is an area of active investigation.

A phenomenon termed DNA damage-induced transcriptional silencing in cis (DISC) halts transcription around DSBs to prevent collision of the transcriptional machinery with the DNA repair machinery [86,87]. Using an elegant Fok1-based reporter system, we showed that mammalian HDACs function as transcriptional repressors even during repair. DISC is regulated by two histone modifications: H3K27me3 installed by EZH2 and H2AK119 mono-ubiquitination (H2AK119ub1) [88]. H2AK119ub1 marks are installed by the polybromo-associated BAF (PBAF) nucleosome remodeler complex and serve as transcriptional repression marks that act downstream of EZH2 during DNA repair [89,90]. We showed that HDACs control the balance of the histone H3K27 acetyl/methyl switch at DSB sites to maintain DISC signals and DNA repair [91,92]. HDAC inhibition reduces histone H3K27me3 at damage sites during active DNA repair but not the global levels of H3K27me3 in spite of the robust increase in total H3K27ac [91,92]. Whether H2AK119ac is mutually exclusive with H2AK119ub1 and whether mammalian HDACs control the H2AK119ub1/ac switch to cause a timely shut down of transcription during repair are being studied in our lab (Figure 1B).

Class I HDACs, HDAC1, 2, and 3, localize to sites of DNA replication for a number of reasons [85,93,94]. HDAC1 and 2 associates with proliferating cell nuclear antigen (PCNA), a DNA sliding clamp that is a co-factor of DNA polymerase and that is involved in recruitment of various factors to the replication fork [93,95]. HDAC3 associates with RbAp48, a component of the CAF1 complex that deposits newly synthesized histones on chromatin during replication [96]. This suggests that HDAC3 plays a role in chromatin maintenance in addition to controlling the fork velocity [96]. Newly synthesized histones are acetylated at H4K5 and H4K12 prior to deposition on nascent chromatin and need to be deacetylated for compact, mature, nascent chromatin to form, which is one reason why HDAC1 and 2 may associate with PCNA [93]. Additionally, evidence suggests that acetylation of histone 4 at lysines 8 and 12 is necessary for DNA decompaction [97]. Using nascent BrdU-CHIP-Slot, a method developed in our lab [93] and a complementary technology to isolate proteins on nascent DNA (iPOND) [94,98], we showed that loss or inhibition of HDAC1 and 2 results in an increase in histone acetylation marks that are involved in chromatin compaction such as H4K16ac, which is known to cause a reduction in the replication fork velocity [93] (Figure 1C). It is not clear if the chromatin structure in front or behind the progressing fork is modulated by HDACs. An answer to this question will provide insight into the mechanisms of HDAC inhibitors.

We did show that HDAC3 is required for DNA replication in hematopoietic stem cells [99], which suggests that a difference in DNA replication rates in cancer versus normal cells provides a therapeutic window for HDAC inhibitor selectivity. In addition, identification of proteins at stalled replication forks using the iPOND technology has led to a tremendous advancement in our understanding of the control of replication stress-induced mechanisms and the consequences of stalled fork-induced replication stress responses on genome integrity [94,98].

**Figure 2:**
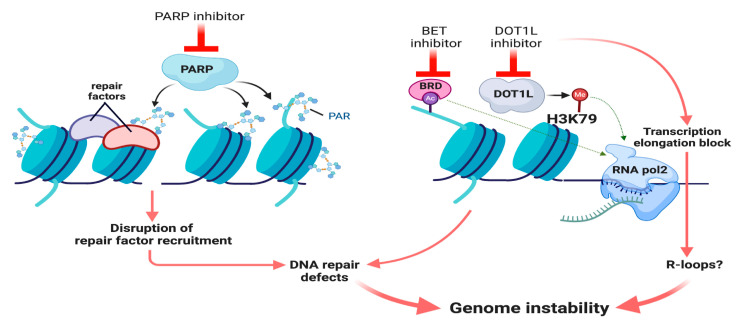
Model for DNA repair- and/or epigenetics- based combinatorial therapies. PARP inhibitor prevents poly-ADP-ribosylation (PAR) of histone or non-histone proteins to disrupt repair factor recruitment. Acetylation (Ac) ‘reader’ Bromodomain (BRD)-containing proteins or DOT1L-mediated H3K79 methylation promote transcription elongation (green dotted line). BET inhibitor or DOT1L inhibitor could impede RNA polymerase II (pol2) progression to cause R-loop formation, or directly disrupt DNA repair. Thus, the combination of these three drugs could effectively compromise genome stability in cancer cells.

In addition to HDACs, other chromatin modifying and remodeler enzymes, such as, SMARCAD1, SMARCA5, DOT1L, BET, HP1, and EZH2 have been associated with the chromatin maintenance during genome integrity [93,94,98,100,101,102,103,104,105,106,107]. Bromodomain and extra-terminal (BET) proteins recognize and bind acetylated lysine residues in histone tails and act as a scaffold for the recruitment of transcription factors and regulators [108]. BET bromodomain proteins have been successfully targeted with JQ1 BET inhibitor (BETi) in a number of cancers including AML which also provides an indirect strategy to target Myc that recruits histone acetyl transferases and co-factors to enhance RNA polymerase II activity [109,110]. BRD4 plays a crucial role in homologous recombination repair pathway, induces BRCAness and increases the sensitivity of solid tumors to PARP inhibitors [111]. Small molecule inhibitors to BET and disruptor of telomeric silencing-like (DOT1L) that primarily participate in transcription and also modulate homologous recombination repair pathway have been recently used along with PARP inhibitors to provide an avenue to increase chemosensitivity in hard-to-treat liquid and solid tumors [112,113,114] (Figure 2).

DOT1L, a non-SET domain methyltransferase, catalyzes the mono, di and trimethylation of H3K79 [115]. DOT1L co-purifies with RNA polymerase II-associated transcriptional elongation complexes [116]. DOT1L-mediated H3K79 methylation (me) is positively correlated with a high transcription elongation rate [117]. Thus, disrupting transcription elongation using DOT1L inhibition or when combined with other transcription inhibitors, such as BETi JQ1, could trigger R-loops and genome instability in cancer cells (Figure 2). DOT1L is highly expressed in GBM and a crucial factor regulating stemness and proliferation of GBM stem cells (GSCs) [118]. Therefore, DOT1L inhibition alone or when combined with standard-of-care GBM therapies, such as alkylating agent temozolomide, could effectively eradicate GBM stem cells. Repair factor 53BP1 functions in checkpoint activation and is recruited to sites of DNA damage through binding of its Tudor domain to the methylated histone residues [119,120]. Although H3K79me is implicated in 53BP1 recruitment, evidence is controversial in mammalian cells, as H4K20me2 and not H3K79me3 is reported to be important for 53BP1 recruitment to damage sites in mammalian cells [121,122]. Nevertheless, both DOT1L and H3K79me are linked to DNA damage repair and genome stability [116,123,124]. A link between H3K79me and DNA resection during HR repair is established and thus, knockdown of DOT1L increases the sensitivity of cancer cells to irradiation and PARP inhibitors, [125]. However, the underlying molecular mechanism(s) remain to be determined and could involve altered chromatin structure, which can then adversely affect DNA repair and transcription [126]. Overall, DOT1L inhibition as a mono or combination therapy holds promise to overcome DNA repair “addiction” and/or cancer stemness.

## 8. Conclusions

Overall, in this review we discuss and propose mechanisms and ideas for DNA repair inhibition and the inhibition of chromatin modifying enzymes with the potential to cause an impact on genome maintenance at multiple levels. This cross talk also connects DNA repair to transcription and replication cellular processes. Because systems are redundant and pathway activation can restore targeted molecular mechanisms inhibited by drugs, therapies associated with various treatments seem to be the most effective strategy to prevent tumor resistance. Targeting DNA repair inhibitors and/or chromatin modifiers should cause sustained high levels or irreversible DNA damage in the tumor cells sufficient to promote efficient death and open the promising tri-therapy to treat cancers. For example, associating a DNA damaging treatment such as radiotherapy to treatments altering DNA repair directly, such as broad-spectrum DNA repair inhibitor AsiDNA, or indirectly, through chromatin remodeling with HDAC inhibitors, should ensure enough lethal damage and the prevention of their repair to allow efficient tumor control with limited toxicity in healthy tissues. These three treatments have already been tested in association two by two (radiotherapy and AsiDNA; NCT01469455, radiotherapy and HDACi; NCT02137759) in clinical trials and have shown good safety profiles but only a moderate efficacy. One can expect that the triple combination would provide sufficient DNA repair inhibition to overcome tumor resistance to radiation.

## Figures and Tables

**Table 1:** List of DNA repair inhibitors that target the double strand break system and their utility in cancers.

Target	Drugs	Side Effects (Clinical Trial Identifier if Applicable)
ATM	AZD1390, KU-59403	AZD1390—seizures, lung problems, muscle pain, dark urine, irregular heartbeat, low blood pressure, light sensitivity, pancreatic or abdominal pain, drop in blood cells counts, increased risk of infection and bleeding problems (NCT03423618) KU-59403—no major toxicity in mice, no clinical trials to date [57]
ATR	AZD6738, VX-970 (M6620), schisandrin B	AZD6738—fatigue, anemia, thrombocytopenia, nausea (NCT02223923) [58] VX-970—no dose-limiting toxicities reported to date in phase I (NCT02487095) [59] schisandrin B—gastric distress, reduced appetite when used as a health supplement, no clinical trial data [60]
PARP	rucaparib, olaparib, niraparib	Similar for all—fatigue, nausea and vomiting, anemia, constipation, increased cholesterol, liver and kidney problems, diarrhea, abdominal pain, decreased appetite (NCT00753545, NCT02655016 and many more) [61,62]
CHK1/CHK2	UCN-01, MK-8776, AZD7762, LY2603618	UCN-01—nausea, vomiting, hypotension, hyperglycemia; has not moved past phase II trials due to low specificity (NCT00003289) [63] MK-8776—QTc prolongation, fatigue, nausea, constipation (NCT00779584) [64] AZD7762—failed phase I clinical trial due to cardiac toxicity (NCT00413686) [46] LY2603618—fatigue, decreased platelets, nausea, decreased neutrophils, decreased hemoglobin; has not moved past phase II as it was not more effective than standard of care (NCT01341457) [65]
DNA-PK	VX-984, M3814, AZD7648, avadomide (CC-122)	VX-984—no data available from ongoing phase I trial (NCT02644278) M3814—fatigue, nausea, constipation, vomiting, decreased appetite, dysphagia, back and chest pain, diarrhea, mucosal inflammation (NCT02516813) AZD7648—no dose-limiting toxicity reported to date in ongoing phase I trial (NCT03907969) avadomide—fatigue, neutropenia, diarrhea [39]
MRN (MRE11)	Mirin	no side effects reported in mice [56]; no human trials have begun
DNA damage response (DDR) Pathways	AsiDNA (DT01)	no side effects yet reported in mice; in humans’ reversible grade 1 and 2 injection site reactions observed but no dose limiting toxicity in ongoing trials (NCT01469455, NCT03579628) [66,67,68,69,70]

## References

[1] Hanahan D., Weinberg R.A. (2011). Hallmarks of cancer: The next generation. Cell.

[2] Kitano K. (2014). Structural mechanisms of human RecQ helicases WRN and BLM. Front. Genet..

[3] Goto M. (2000). Werner’s syndrome: From clinics to genetics. Clin. Exp. Rheumatol..

[4] Manthei K.A., Keck J.L. (2013). The BLM dissolvasome in DNA replication and repair. Cell. Mol. Life Sci..

[5] German J. (1995). Bloom’s syndrome. Dermatol. Clin..

[6] Aggarwal M., Sommers J.A., Shoemaker R.H., Brosh R.M. (2011). Inhibition of helicase activity by a small molecule impairs Werner syndrome helicase (WRN) function in the cellular response to DNA damage or replication stress. Proc. Natl. Acad. Sci. USA.

[7] Hengel S.R., Spies M.A., Spies M. (2017). Small-Molecule Inhibitors Targeting DNA Repair and DNA Repair Deficiency in Research and Cancer Therapy. Cell Chem. Biol..

[8] Mirzoeva O.K., Petrini J.H. (2001). DNA damage-dependent nuclear dynamics of the Mre11 complex. Mol. Cell. Biol..

[9] Kastan M.B. (2008). DNA damage responses: Mechanisms and roles in human disease: 2007 G.H.A. Clowes Memorial Award Lecture. Mol. Cancer Res..

[10] O’Driscoll M. (2012). Diseases associated with defective responses to DNA damage. Cold Spring Harb. Perspect. Biol..

[11] Spycher C., Miller E.S., Townsend K., Pavic L., Morrice N.A., Janscak P., Stewart G.S., Stucki M. (2008). Constitutive phosphorylation of MDC1 physically links the MRE11-RAD50-NBS1 complex to damaged chromatin. J. Cell Biol..

[12] Nakanishi K., Taniguchi T., Ranganathan V., New H.V., Moreau L.A., Stotsky M., Mathew C.G., Kastan M.B., Weaver D.T., D’Andrea A.D. (2002). Interaction of FANCD2 and NBS1 in the DNA damage response. Nat. Cell Biol..

[13] Waltes R., Kalb R., Gatei M., Kijas A.W., Stumm M., Sobeck A., Wieland B., Varon R., Lerenthal Y., Lavin M.F. (2009). Human RAD50 deficiency in a Nijmegen breakage syndrome-like disorder. Am. J. Hum. Genet..

[14] Lavin M.F. (2008). Ataxia-telangiectasia: From a rare disorder to a paradigm for cell signalling and cancer. Nat. Rev. Mol. Cell. Biol..

[15] Lavin M.F. (2007). ATM and the Mre11 complex combine to recognize and signal DNA double-strand breaks. Oncogene.

[16] Huang R.X., Zhou P.K. (2020). DNA damage response signaling pathways and targets for radiotherapy sensitization in cancer. Signal Transduct. Target. Ther..

[17] Durant S.T., Zheng L., Wang Y., Chen K., Zhang L., Zhang T., Yang Z., Riches L., Trinidad A.G., Fok J.H.L. (2018). The brain-penetrant clinical ATM inhibitor AZD1390 radiosensitizes and improves survival of preclinical brain tumor models. Sci. Adv..

[18] Shiotani B., Zou L. (2009). ATR signaling at a glance. J. Cell Sci..

[19] Ball H.L., Myers J.S., Cortez D. (2005). ATRIP binding to replication protein A-single-stranded DNA promotes ATR-ATRIP localization but is dispensable for Chk1 phosphorylation. Mol. Biol. Cell.

[20] Vassin V.M., Anantha R.W., Sokolova E., Kanner S., Borowiec J.A. (2009). Human RPA phosphorylation by ATR stimulates DNA synthesis and prevents ssDNA accumulation during DNA-replication stress. J. Cell Sci..

[21] Ma M., Rodriguez A., Sugimoto K. (2020). Activation of ATR-related protein kinase upon DNA damage recognition. Curr. Genet..

[22] Ubhi T., Brown G.W. (2019). Exploiting DNA Replication Stress for Cancer Treatment. Cancer Res..

[23] Tanaka A., Weinel S., Nagy N., O’Driscoll M., Lai-Cheong J.E., Kulp-Shorten C.L., Knable A., Carpenter G., Fisher S.A., Hiragun M. (2012). Germline mutation in ATR in autosomal- dominant oropharyngeal cancer syndrome. Am. J. Hum. Genet..

[24] Lewis K.A., Mullany S., Thomas B., Chien J., Loewen R., Shridhar V., Cliby W.A. (2005). Heterozygous ATR mutations in mismatch repair-deficient cancer cells have functional significance. Cancer Res..

[25] Moiseeva T.N., Yin Y., Calderon M.J., Qian C., Schamus-Haynes S., Sugitani N., Osmanbeyoglu H.U., Rothenberg E., Watkins S.C., Bakkenist C.J. (2019). An ATR and CHK1 kinase signaling mechanism that limits origin firing during unperturbed DNA replication. Proc. Natl. Acad. Sci. USA.

[26] Qvist P., Huertas P., Jimeno S., Nyegaard M., Hassan M.J., Jackson S.P., Borglum A.D. (2011). CtIP Mutations Cause Seckel and Jawad Syndromes. PLoS Genet..

[27] O’Driscoll M., Ruiz-Perez V.L., Woods C.G., Jeggo P.A., Goodship J.A. (2003). A splicing mutation affecting expression of ataxia-telangiectasia and Rad3-related protein (ATR) results in Seckel syndrome. Nat. Genet..

[28] Alderton G.K., Joenje H., Varon R., Borglum A.D., Jeggo P.A., O’Driscoll M. (2004). Seckel syndrome exhibits cellular features demonstrating defects in the ATR-signalling pathway. Hum. Mol. Genet..

[29] Yazinski S.A., Zou L. (2016). Functions, Regulation, and Therapeutic Implications of the ATR Checkpoint Pathway. Annu. Rev. Genet..

[30] Marechal A., Zou L. (2013). DNA damage sensing by the ATM and ATR kinases. Cold Spring Harb. Perspect. Biol..

[31] Yang J., Xu Z.P., Huang Y., Hamrick H.E., Duerksen-Hughes P.J., Yu Y.N. (2004). ATM and ATR: Sensing DNA damage. World J. Gastroenterol..

[32] Brown E.J., Baltimore D. (2000). ATR disruption leads to chromosomal fragmentation and early embryonic lethality. Genes Dev..

[33] Ward I.M., Chen J. (2001). Histone H2AX is phosphorylated in an ATR-dependent manner in response to replicational stress. J. Biol. Chem..

[34] Shiotani B., Zou L. (2009). Single-stranded DNA orchestrates an ATM-to-ATR switch at DNA breaks. Mol. Cell.

[35] Tomimatsu N., Mukherjee B., Burma S. (2009). Distinct roles of ATR and DNA-PKcs in triggering DNA damage responses in ATM-deficient cells. EMBO Rep..

[36] Chanoux R.A., Yin B., Urtishak K.A., Asare A., Bassing C.H., Brown E.J. (2009). ATR and H2AX cooperate in maintaining genome stability under replication stress. J. Biol. Chem..

[37] Goodwin J.F., Knudsen K.E. (2014). Beyond DNA repair: DNA-PK function in cancer. Cancer Discov..

[38] Davidson D., Amrein L., Panasci L., Aloyz R. (2013). Small Molecules, Inhibitors of DNA-PK, Targeting DNA Repair, and Beyond. Front. Pharmacol..

[39] Rasco D.W., Papadopoulos K.P., Pourdehnad M., Gandhi A.K., Hagner P.R., Li Y., Wei X., Chopra R., Hege K., DiMartino J. (2019). A First-in-Human Study of Novel Cereblon Modulator Avadomide (CC-122) in Advanced Malignancies. Clin. Cancer Res..

[40] Carr M.I., Zimmermann A., Chiu L.Y., Zenke F.T., Blaukat A., Vassilev L.T. (2020). DNA-PK Inhibitor, M3814, as a New Combination Partner of Mylotarg in the Treatment of Acute Myeloid Leukemia. Front. Oncol..

[41] Wise H.C., Iyer G.V., Moore K., Temkin S.M., Gordon S., Aghajanian C., Grisham R.N. (2019). Activity of M3814, an Oral DNA-PK Inhibitor, In Combination with Topoisomerase II Inhibitors in Ovarian Cancer Models. Sci. Rep..

[42] Liu Q., Guntuku S., Cui X.S., Matsuoka S., Cortez D., Tamai K., Luo G., Carattini-Rivera S., DeMayo F., Bradley A. (2000). Chk1 is an essential kinase that is regulated by Atr and required for the G(2)/M DNA damage checkpoint. Genes Dev..

[43] Molinari M. (2000). Cell cycle checkpoints and their inactivation in human cancer. Cell Prolif..

[44] Zhang Y., Hunter T. (2014). Roles of Chk1 in cell biology and cancer therapy. Int. J. Cancer.

[45] Visconti R., Della Monica R., Grieco D. (2016). Cell cycle checkpoint in cancer: A therapeutically targetable double-edged sword. J. Exp. Clin. Cancer Res..

[46] Sausville E., Lorusso P., Carducci M., Carter J., Quinn M.F., Malburg L., Azad N., Cosgrove D., Knight R., Barker P. (2014). Phase I dose-escalation study of AZD7762, a checkpoint kinase inhibitor, in combination with gemcitabine in US patients with advanced solid tumors. Cancer Chemother. Pharmacol..

[47] Tang Y., Dai Y., Grant S., Dent P. (2012). Enhancing CHK1 inhibitor lethality in glioblastoma. Cancer Biol. Ther..

[48] Haince J.F., McDonald D., Rodrigue A., Dery U., Masson J.Y., Hendzel M.J., Poirier G.G. (2008). PARP1-dependent kinetics of recruitment of MRE11 and NBS1 proteins to multiple DNA damage sites. J. Biol. Chem..

[49] Malyuchenko N.V., Kotova E.Y., Kulaeva O.I., Kirpichnikov M.P., Studitskiy V.M. (2015). PARP1 Inhibitors: Antitumor drug design. Acta Nat..

[50] Roy R., Chun J., Powell S.N. (2011). BRCA1 and BRCA2: Different roles in a common pathway of genome protection. Nat. Rev. Cancer.

[51] Li H., Liu Z.Y., Wu N., Chen Y.C., Cheng Q., Wang J. (2020). PARP inhibitor resistance: The underlying mechanisms and clinical implications. Mol. Cancer.

[52] Lord C.J., Ashworth A. (2017). PARP inhibitors: Synthetic lethality in the clinic. Science.

[53] Jannetti S.A., Zeglis B.M., Zalutsky M.R., Reiner T. (2020). Poly(ADP-Ribose)Polymerase (PARP) Inhibitors and Radiation Therapy. Front. Pharmacol..

[54] Coleman R.L., Oza A.M., Lorusso D., Aghajanian C., Oaknin A., Dean A., Colombo N., Weberpals J.I., Clamp A., Scambia G. (2017). Rucaparib maintenance treatment for recurrent ovarian carcinoma after response to platinum therapy (ARIEL3): A randomised, double-blind, placebo-controlled, phase 3 trial. Lancet.

[55] Karnitz L.M., Zou L. (2015). Molecular Pathways: Targeting ATR in Cancer Therapy. Clin. Cancer Res..

[56] Petroni M., Sardina F., Infante P., Bartolazzi A., Locatelli E., Fabretti F., Di Giulio S., Capalbo C., Cardinali B., Coppa A. (2018). MRE11 inhibition highlights a replication stress-dependent vulnerability of MYCN-driven tumors. Cell Death Dis..

[57] Batey M.A., Zhao Y., Kyle S., Richardson C., Slade A., Martin N.M., Lau A., Newell D.R., Curtin N.J. (2013). Preclinical evaluation of a novel ATM inhibitor, KU59403, in vitro and in vivo in p53 functional and dysfunctional models of human cancer. Mol. Cancer Ther..

[58] Dillon M.T., Boylan Z., Smith D., Guevara J., Mohammed K., Peckitt C., Saunders M., Banerji U., Clack G., Smith S.A. (2018). PATRIOT: A phase I study to assess the tolerability, safety and biological effects of a specific ataxia telangiectasia and Rad3-related (ATR) inhibitor (AZD6738) as a single agent and in combination with palliative radiation therapy in patients with solid tumours. Clin. Transl. Radiat. Oncol..

[59] Yap T.A., O’Carrigan B., Penney M.S., Lim J.S., Brown J.S., de Miguel Luken M.J., Tunariu N., Perez-Lopez R., Rodrigues D.N., Riisnaes R. (2020). Phase I Trial of First-in-Class ATR Inhibitor M6620 (VX-970) as Monotherapy or in Combination With Carboplatin in Patients With Advanced Solid Tumors. J. Clin. Oncol..

[60] Nasser M.I., Zhu S., Chen C., Zhao M., Huang H., Zhu P. (2020). A Comprehensive Review on Schisandrin B and Its Biological Properties. Oxid. Med. Cell. Longev..

[61] Evans T., Matulonis U. (2017). PARP inhibitors in ovarian cancer: Evidence, experience and clinical potential. Ther. Adv. Med. Oncol..

[62] Brown J.S., Kaye S.B., Yap T.A. (2016). PARP inhibitors: The race is on. Br. J. Cancer.

[63] Dees E.C., Baker S.D., O’Reilly S., Rudek M.A., Davidson S.B., Aylesworth C., Elza-Brown K., Carducci M.A., Donehower R.C. (2005). A phase I and pharmacokinetic study of short infusions of UCN-01 in patients with refractory solid tumors. Clin. Cancer Res..

[64] Daud A.I., Ashworth M.T., Strosberg J., Goldman J.W., Mendelson D., Springett G., Venook A.P., Loechner S., Rosen L.S., Shanahan F. (2015). Phase I dose-escalation trial of checkpoint kinase 1 inhibitor MK-8776 as monotherapy and in combination with gemcitabine in patients with advanced solid tumors. J. Clin. Oncol..

[65] Calvo E., Braiteh F., Von Hoff D., McWilliams R., Becerra C., Galsky M.D., Jameson G., Lin J., McKane S., Wickremsinhe E.R. (2016). Phase I Study of CHK1 Inhibitor LY2603618 in Combination with Gemcitabine in Patients with Solid Tumors. Oncology.

[66] Biau J., Devun F., Jdey W., Kotula E., Quanz M., Chautard E., Sayarath M., Sun J.S., Verrelle P., Dutreix M. (2014). A preclinical study combining the DNA repair inhibitor Dbait with radiotherapy for the treatment of melanoma. Neoplasia.

[67] Biau J., Devun F., Verrelle P., Dutreix M. (2016). Dbait: An innovative concept to inhibit DNA repair and treat cancer. Bull. Cancer.

[68] Biau J., Chautard E., Berthault N., de Koning L., Court F., Pereira B., Verrelle P., Dutreix M. (2019). Combining the DNA Repair Inhibitor Dbait With Radiotherapy for the Treatment of High Grade Glioma: Efficacy and Protein Biomarkers of Resistance in Preclinical Models. Front. Oncol..

[69] Ferreira S., Dutreix M. (2019). DNA repair inhibitors to enhance radiotherapy: Progresses and limitations. Cancer Radiother..

[70] Ferreira S., Foray C., Gatto A., Larcher M., Heinrich S., Lupu M., Mispelter J., Boussin F.D., Pouponnot C., Dutreix M. (2020). AsiDNA Is a Radiosensitizer with no Added Toxicity in Medulloblastoma Pediatric Models. Clin. Cancer Res..

[71] Le Tourneau C., Dreno B., Kirova Y., Grob J.J., Jouary T., Dutriaux C., Thomas L., Lebbe C., Mortier L., Saiag P. (2016). First-in-human phase I study of the DNA-repair inhibitor DT01 in combination with radiotherapy in patients with skin metastases from melanoma. Br. J. Cancer.

[72] Devun F., Bousquet G., Biau J., Herbette A., Roulin C., Berger F., Sun J.S., Robine S., Dutreix M. (2012). Preclinical study of the DNA repair inhibitor Dbait in combination with chemotherapy in colorectal cancer. J. Gastroenterol..

[73] Horwitz S.B. (1994). Taxol (paclitaxel): Mechanisms of action. Ann. Oncol..

[74] Dasari S., Tchounwou P.B. (2014). Cisplatin in cancer therapy: Molecular mechanisms of action. Eur. J. Pharmacol..

[75] Zhu Q., Wani A.A. (2010). Histone modifications: Crucial elements for damage response and chromatin restoration. J. Cell. Physiol..

[76] Soria G., Polo S.E., Almouzni G. (2012). Prime, repair, restore: The active role of chromatin in the DNA damage response. Mol. Cell.

[77] Tjeertes J.V., Miller K.M., Jackson S.P. (2009). Screen for DNA-damage-responsive histone modifications identifies H3K9Ac and H3K56Ac in human cells. EMBO J..

[78] Xie W., Song C., Young N.L., Sperling A.S., Xu F., Sridharan R., Conway A.E., Garcia B.A., Plath K., Clark A.T. (2009). Histone h3 lysine 56 acetylation is linked to the core transcriptional network in human embryonic stem cells. Mol. Cell.

[79] Hamperl S., Bocek M.J., Saldivar J.C., Swigut T., Cimprich K.A. (2017). Transcription-Replication Conflict Orientation Modulates R-Loop Levels and Activates Distinct DNA Damage Responses. Cell.

[80] Crossley M.P., Bocek M., Cimprich K.A. (2019). R-Loops as Cellular Regulators and Genomic Threats. Mol. Cell.

[81] Allison D.F., Wang G.G. (2019). R-loops: Formation, function, and relevance to cell stress. Cell Stress.

[82] Sollier J., Cimprich K.A. (2015). Breaking bad: R-loops and genome integrity. Trends Cell Biol..

[83] Rodriguez A., D’Andrea A. (2017). Fanconi anemia pathway. Curr. Biol..

[84] Zhao H., Zhu M., Limbo O., Russell P. (2018). RNase H eliminates R-loops that disrupt DNA replication but is nonessential for efficient DSB repair. EMBO Rep..

[85] Tharkar-Promod S., Johnson D.P., Bennett S.E., Dennis E.M., Banowsky B.G., Jones S.S., Shearstone J.R., Quayle S.N., Min C., Jarpe M. (2018). HDAC1,2 inhibition and doxorubicin impair Mre11-dependent DNA repair and DISC to override BCR-ABL1-driven DSB repair in Philadelphia chromosome-positive B-cell precursor acute lymphoblastic leukemia. Leukemia.

[86] Kakarougkas A., Downs J.A., Jeggo P.A. (2015). The PBAF chromatin remodeling complex represses transcription and promotes rapid repair at DNA double-strand breaks. Mol. Cell. Oncol..

[87] Clouaire T., Legube G. (2019). A Snapshot on the Cis Chromatin Response to DNA Double-Strand Breaks. Trends Genet..

[88] Shanbhag N.M., Rafalska-Metcalf I.U., Balane-Bolivar C., Janicki S.M., Greenberg R.A. (2010). ATM-dependent chromatin changes silence transcription in cis to DNA double-strand breaks. Cell.

[89] Kakarougkas A., Ismail A., Chambers A.L., Riballo E., Herbert A.D., Kunzel J., Lobrich M., Jeggo P.A., Downs J.A. (2014). Requirement for PBAF in transcriptional repression and repair at DNA breaks in actively transcribed regions of chromatin. Mol. Cell.

[90] Vissers J.H., van Lohuizen M., Citterio E. (2012). The emerging role of Polycomb repressors in the response to DNA damage. J. Cell Sci..

[91] Johnson D.P., Spitz G.S., Tharkar S., Quayle S.N., Shearstone J.R., Jones S., McDowell M.E., Wellman H., Tyler J.K., Cairns B.R. (2015). HDAC1,2 inhibition impairs EZH2- and BBAP-mediated DNA repair to overcome chemoresistance in EZH2 gain-of-function mutant diffuse large B-cell lymphoma. Oncotarget.

[92] Johnson D.P., Spitz-Becker G.S., Chakraborti K., Bhaskara S. (2019). Assessment of epigenetic mechanisms and DNA double-strand break repair using laser micro-irradiation technique developed for hematological cells. EBioMedicine.

[93] Bhaskara S., Jacques V., Rusche J.R., Olson E.N., Cairns B.R., Chandrasekharan M.B. (2013). Histone deacetylases 1 and 2 maintain S-phase chromatin and DNA replication fork progression. Epigenet. Chromatin.

[94] Sirbu B.M., Couch F.B., Feigerle J.T., Bhaskara S., Hiebert S.W., Cortez D. (2011). Analysis of protein dynamics at active, stalled, and collapsed replication forks. Genes Dev..

[95] Moldovan G.L., Pfander B., Jentsch S. (2007). PCNA, the maestro of the replication fork. Cell.

[96] Wells C.E., Bhaskara S., Stengel K.R., Zhao Y., Sirbu B., Chagot B., Cortez D., Khabele D., Chazin W.J., Cooper A. (2013). Inhibition of histone deacetylase 3 causes replication stress in cutaneous T cell lymphoma. PLoS ONE.

[97] Ruan K., Yamamoto T.G., Asakawa H., Chikashige Y., Kimura H., Masukata H., Haraguchi T., Hiraoka Y. (2015). Histone H4 acetylation required for chromatin decompaction during DNA replication. Sci. Rep..

[98] Sirbu B.M., McDonald W.H., Dungrawala H., Badu-Nkansah A., Kavanaugh G.M., Chen Y., Tabb D.L., Cortez D. (2013). Identification of proteins at active, stalled, and collapsed replication forks using isolation of proteins on nascent DNA (iPOND) coupled with mass spectrometry. J. Biol. Chem..

[99] Summers A.R., Fischer M.A., Stengel K.R., Zhao Y., Kaiser J.F., Wells C.E., Hunt A., Bhaskara S., Luzwick J.W., Sampathi S. (2013). HDAC3 is essential for DNA replication in hematopoietic progenitor cells. J. Clin. Investig..

[100] Rondinelli B., Gogola E., Yucel H., Duarte A.A., van de Ven M., van der Sluijs R., Konstantinopoulos P.A., Jonkers J., Ceccaldi R., Rottenberg S. (2017). EZH2 promotes degradation of stalled replication forks by recruiting MUS81 through histone H3 trimethylation. Nat. Cell Biol..

[101] Quivy J.P., Gerard A., Cook A.J., Roche D., Almouzni G. (2008). The HP1-p150/CAF-1 interaction is required for pericentric heterochromatin replication and S-phase progression in mouse cells. Nat. Struct. Mol. Biol..

[102] Collins N., Poot R.A., Kukimoto I., Garcia-Jimenez C., Dellaire G., Varga-Weisz P.D. (2002). An ACF1-ISWI chromatin-remodeling complex is required for DNA replication through heterochromatin. Nat. Genet..

[103] Fu H., Maunakea A.K., Martin M.M., Huang L., Zhang Y., Ryan M., Kim R., Lin C.M., Zhao K., Aladjem M.I. (2013). Methylation of histone H3 on lysine 79 associates with a group of replication origins and helps limit DNA replication once per cell cycle. PLoS Genet..

[104] Unterberger A., Andrews S.D., Weaver I.C., Szyf M. (2006). DNA methyltransferase 1 knockdown activates a replication stress checkpoint. Mol. Cell. Biol..

[105] Cortez D. (2017). Proteomic Analyses of the Eukaryotic Replication Machinery. Methods Enzymol..

[106] Bhaskara S., Knutson S.K., Jiang G., Chandrasekharan M.B., Wilson A.J., Zheng S., Yenamandra A., Locke K., Yuan J.L., Bonine-Summers A.R. (2010). Hdac3 is essential for the maintenance of chromatin structure and genome stability. Cancer Cell.

[107] Bhaskara S., Hiebert S.W. (2011). Role for histone deacetylase 3 in maintenance of genome stability. Cell Cycle.

[108] Bhattacharya S., Piya S., Borthakur G. (2018). Bromodomain inhibitors: What does the future hold?. Clin. Adv. Hematol. Oncol..

[109] Yang L., Zhang Y., Shan W., Hu Z., Yuan J., Pi J., Wang Y., Fan L., Tang Z., Li C. (2017). Repression of BET activity sensitizes homologous recombination-proficient cancers to PARP inhibition. Sci. Transl. Med..

[110] Pericole F.V., Lazarini M., de Paiva L.B., Duarte A., Vieira Ferro K.P., Niemann F.S., Roversi F.M., Olalla Saad S.T. (2019). BRD4 Inhibition Enhances Azacitidine Efficacy in Acute Myeloid Leukemia and Myelodysplastic Syndromes. Front. Oncol..

[111] Mio C., Gerratana L., Bolis M., Caponnetto F., Zanello A., Barbina M., Di Loreto C., Garattini E., Damante G., Puglisi F. (2019). BET proteins regulate homologous recombination-mediated DNA repair: BRCAness and implications for cancer therapy. Int. J. Cancer.

[112] Muvarak N.E., Chowdhury K., Xia L., Robert C., Choi E.Y., Cai Y., Bellani M., Zou Y., Singh Z.N., Duong V.H. (2016). Enhancing the Cytotoxic Effects of PARP Inhibitors with DNA Demethylating Agents—A Potential Therapy for Cancer. Cancer Cell.

[113] Maifrede S., Martinez E., Nieborowska-Skorska M., Di Marcantonio D., Hulse M., Le B.V., Zhao H., Piwocka K., Tempera I., Sykes S.M. (2017). MLL-AF9 leukemias are sensitive to PARP1 inhibitors combined with cytotoxic drugs. Blood Adv..

[114] Karakashev S., Zhu H., Yokoyama Y., Zhao B., Fatkhutdinov N., Kossenkov A.V., Wilson A.J., Simpkins F., Speicher D., Khabele D. (2017). BET Bromodomain Inhibition Synergizes with PARP Inhibitor in Epithelial Ovarian Cancer. Cell Rep..

[115] Wong M., Polly P., Liu T. (2015). The histone methyltransferase DOT1L: Regulatory functions and a cancer therapy target. Am. J. Cancer Res..

[116] Wood K., Tellier M., Murphy S. (2018). DOT1L and H3K79 Methylation in Transcription and Genomic Stability. Biomolecules.

[117] Veloso A., Kirkconnell K.S., Magnuson B., Biewen B., Paulsen M.T., Wilson T.E., Ljungman M. (2014). Rate of elongation by RNA polymerase II is associated with specific gene features and epigenetic modifications. Genome Res..

[118] MacLeod G., Bozek D.A., Rajakulendran N., Monteiro V., Ahmadi M., Steinhart Z., Kushida M.M., Yu H., Coutinho F.J., Cavalli F.M.G. (2019). Genome-Wide CRISPR-Cas9 Screens Expose Genetic Vulnerabilities and Mechanisms of Temozolomide Sensitivity in Glioblastoma Stem Cells. Cell Rep..

[119] Dai Y., Zhang A., Shan S., Gong Z., Zhou Z. (2018). Structural basis for recognition of 53BP1 tandem Tudor domain by TIRR. Nat. Commun..

[120] Zgheib O., Pataky K., Brugger J., Halazonetis T.D. (2009). An oligomerized 53BP1 tudor domain suffices for recognition of DNA double-strand breaks. Mol. Cell. Biol..

[121] Botuyan M.V., Lee J., Ward I.M., Kim J.E., Thompson J.R., Chen J., Mer G. (2006). Structural basis for the methylation state-specific recognition of histone H4-K20 by 53BP1 and Crb2 in DNA repair. Cell.

[122] Paquin K.L., Howlett N.G. (2018). Understanding the Histone DNA Repair Code: H4K20me2 Makes Its Mark. Mol. Cancer Res..

[123] Kari V., Raul S.K., Henck J.M., Kitz J., Kramer F., Kosinsky R.L., Ubelmesser N., Mansour W.Y., Eggert J., Spitzner M. (2019). The histone methyltransferase DOT1L is required for proper DNA damage response, DNA repair, and modulates chemotherapy responsiveness. Clin. Epigenet..

[124] Zhu B., Chen S., Wang H., Yin C., Han C., Peng C., Liu Z., Wan L., Zhang X., Zhang J. (2018). The protective role of DOT1L in UV-induced melanomagenesis. Nat. Commun..

[125] Liu W., Deng L., Song Y., Redell M. (2014). DOT1L inhibition sensitizes MLL-rearranged AML to chemotherapy. PLoS ONE.

[126] Oksenych V., Zhovmer A., Ziani S., Mari P.O., Eberova J., Nardo T., Stefanini M., Giglia-Mari G., Egly J.M., Coin F. (2013). Histone methyltransferase DOT1L drives recovery of gene expression after a genotoxic attack. PLoS Genet..

